# Target product profile for a test for the early assessment of treatment efficacy in Chagas disease patients: An expert consensus

**DOI:** 10.1371/journal.pntd.0008035

**Published:** 2020-04-23

**Authors:** Julio Alonso-Padilla, Marcelo Abril, Belkisyolé Alarcón de Noya, Igor C. Almeida, Andrea Angheben, Tania Araujo Jorge, Eric Chatelain, Monica Esteva, Joaquim Gascón, Mario J. Grijalva, Felipe Guhl, Alejandro Marcel Hasslocher-Moreno, Manuel Carlos López, Alejandro Luquetti, Oscar Noya, María Jesús Pinazo, Janine M. Ramsey, Isabela Ribeiro, Andres Mariano Ruiz, Alejandro G. Schijman, Sergio Sosa-Estani, M. Carmen Thomas, Faustino Torrico, Maan Zrein, Albert Picado

**Affiliations:** 1 Barcelona Institute for Global Health (ISGlobal), Hospital Clínic—University of Barcelona, Barcelona, Spain; 2 Fundación Mundo Sano, Buenos Aires, Argentina; 3 Instituto de Medicina Tropical, Universidad Central de Venezuela, Caracas, Venezuela; 4 Border Biomedical Research Center, Department of Biological Sciences, University of Texas at El Paso, El Paso, Texas, United States of America; 5 Department of Infectious–Tropical Diseases and Microbiology, IRCCS Sacro Cuore Don Calabria Hospital, Negrar di Valpolicella, Verona, Italy; 6 Instituto Oswaldo Cruz, Fundaçao Instituto Oswaldo Cruz, Rio de Janeiro, Brazil; 7 Drugs for Neglected Diseases Initiative (DNDi), Geneva, Switzerland; 8 Instituto Nacional de Parasitología “Dr. Mario Fatala Chaben”, ANLIS “Dr. Carlos G. Malbrán”, Ministerio de Salud, Buenos Aires, Argentina; 9 Centro de Investigación para la Salud en América Latina (CISeAL), Pontificia Universidad Católica del Ecuador, Quito, Ecuador; 10 Infectious and Tropical Disease Institute, Biomedical Sciences Department, Ohio University, Athens, Ohio, United States of America; 11 Centro de Investigaciones en Microbiología y Parasitología Tropical (CIMPAT), Universidad de los Andes, Bogotá, Colombia; 12 Instituto Nacional de Infectologia Evandro Chagas (INI), Fundação Oswaldo Cruz—Ministério da Saúde, Brazil; 13 Instituto de Parasitología y Biomedicina López Neyra (IPBLN), Consejo Superior de Investigaciones Científicas (CSIC), Granada, Spain; 14 Hospital das Clínicas, Federal University of Goiás, Goiania, Brazil; 15 Instituto Nacional de Salud Pública/CRISP, Tapachula, Chiapas, Mexico; 16 Instituto de Investigaciones en Ingeniería Genética y Biología Molecular (INGEBI), Consejo Nacional de Investigaciones Científicas y Técnicas (CONICET), Buenos Aires, Argentina; 17 Drugs for Neglected Disease initiative (DNDi) Latin America, Rio de Janeiro, Brazil; 18 Epidemiology and Public Health Research Center, CONICET, Buenos Aires, Argentina; 19 Fundación CEADES; Universidad Mayor de San Simón, Cochabamba, Bolivia; 20 Infynity Biomarkers, Lyon, France; 21 Foundation for Innovative Diagnostics (FIND), Geneva, Switzerland; Universidade Federal de Minas Gerais, BRAZIL

## Introduction

Six to 7 million people are estimated to be infected by *Trypanosoma cruzi*, the parasite causing Chagas disease [1]. Thirty to 40% of them, i.e., 1.8 to 2.4 million people, will suffer cardiac disorders and/or digestive clinical manifestations if they are not treated early during the course of the infection [1, 2]. However, only a small fraction of patients are properly diagnosed and treated [3]. Current clinical guidelines recommend treating *T*. *cruzi*–infected people if they are asymptomatic or present early symptoms of the disease (Table 1) [4, 5]. Benznidazole (BNZ) and nifurtimox (NFX) are the first-line antiparasitic treatments currently available, both with long administration regimens (60 days) that can produce adverse side effects [6–8]. Despite the fact they are not 100% effective in patients with chronic disease [9–12], they are the only drugs currently registered, and the benefits of their administration have been confirmed in several clinical studies [9–14]. Currently, clinical trials with new compounds, using alternative regimens that aim to maintain efficacy whilst reducing toxicity, are ongoing and could lead to new therapeutic opportunities and/or policy change [15].

**Table 1:** Definitions used to develop the TPP for a test for the early assessment of treatment efficacy in Chagas disease patients.

Concept	Definition	Current diagnostic
**Acute Chagas disease**	The first phase of *T*. *cruzi* infection is characterized by a high number of parasites circulating in the blood that can be detected by direct methods (e.g., microscopy). In most cases, symptoms are absent or mild and unspecific. Acute Chagas disease occurs after a short incubation time (5–15 days on average, longer for cases of transmission by blood transfusion) and can last for 2 months. Infection may occur by vectorial transmission when *T*. *cruzi* parasites enter the body via a skin break caused by a bug bite, by skin breaching after scratching the bite site, or via mucosal entry (e.g., oral transmission through contaminated food). Vector-independent transmission routes include: congenital infection; blood transfusion; cell, blood, or tissue transplantation; and needle sharing. Infection can also occur accidentally after the manipulation of infected triatomines and/or infected animals or laboratory samples. Immunocompromised patients with chronic *T*.* cruzi* infection are at risk of the disease being reactivated and then undergoing an acute presentation with a high mortality rate.	During the acute phase, *T*. *cruzi* infection is diagnosed by direct detection of the parasite or parasite DNA circulating in the bloodstream or the detection of specific IgM and IgG antibodies.
**Chronic Chagas disease**	After a variable period (4–8 months) of infection or after unsuccessful treatment, the chronic phase is established during which *T*. *cruzi* parasites mainly persist in a variety of tissues. Patients in the chronic phase of the disease can be clinically divided into two groups: •**Asymptomatic** patients without demonstrable disease, who are characterized by the absence of damage or organ alterations following evaluation through “classic” diagnostic tools (electrocardiogram, plain thoracic X-rays, echocardiogram, Rezende technique). These patients’ clinical status is also known as the **chronic indeterminate form**. •**Symptomatic** patients with demonstrable disease (around 30%–40% of those chronically infected), who show a variable degree of cardiac disorder and/or digestive clinical manifestations. They suffer from the **chronic determinate form**. Chronic Chagas disease patients can also be classified based on the level of tissue damage (e.g., Kuschnir´s modified classification for cardiac damage or Ximenes and Rezende classifications for digestive damage) [24].	Patients in the chronic phase are diagnosed via the detection of *T*. *cruzi* antibodies which, according to WHO recommendations, entails obtaining concordant positivity in two tests based on different sets of *T*. *cruzi* antigens [4].
**Chagas disease treatment**	According to current guidelines [25], treatment should be offered to all patients except those with advanced Chagas disease (e.g., Kuschnir grade III), where it is not recommended. • In patients with Kuschnir grade II, age can be taken into consideration when evaluating treatment administration. • Treatment of patients with digestive damage is dependent on the degree of involvement, similar to the approach for cardiac patients (not an evidence-based recommendation). Arguments in favor of excluding advanced cases from treatment are based on the rationale that in the late stages of the disease, parasite load and activity may no longer be relevant in determining disease evolution. This was concluded by the BENEFIT trial (ClinicalTrials.gov Identifier: NCT00123916) in relation to cardiac pathology [12].	
**Cure in Chagas disease patients**	Elimination of *T*. *cruzi* parasites from the patient’s body following treatment.	
**Treatment efficacy**	Treatment success: elimination of *T. cruzi* parasites from the patient’s body, independently of whether the infection is asymptomatic or symptomatic, after specific treatment. Treatment failure: the detection of *T. cruzi* parasites in the patient’s body after specific treatment.	Markers of *T*. *cruzi* elimination (treatment success): • Indirect: seroconversion (from positive to negative) in terms of reactivity against *T*. *cruzi* antigens. Markers of *T*. *cruzi* presence (treatment failure): • Direct: positive parasitemia measured by *T*. *cruzi* DNA amplification reaction. • Indirect: persistence of reactivity against *T*. *cruzi* antigens.

IgG, immunoglobulin G; IgM, immunoglobulin M; TPP, target product profile; WHO, World Health Organization

In any case, the absence of a test for the early assessment of treatment efficacy, often called a test of cure (ToC), is a major obstacle to Chagas disease control. Accurately monitoring treatment response would undoubtedly improve patient management and support the conduct of clinical trials. Although treatment efficacy and treatment response may be conceptually different, we are using these terms synonymously for the purpose of the current target product profile (TPP) [16, 17].

Unfortunately, there is no gold-standard test for the early determination of whether someone who has been treated for chronic Chagas disease has been cured or not. Current methods used for monitoring Chagas disease treatment efficacy are suboptimal due to the fact that: (1) clinical progression of the disease is silent and associated with complex and mostly unknown host–pathogen interactions; (2) once in the chronic stage, infected subjects remain seropositive for years, with very low and intermittent parasitemia counts; and (3) as a consequence, in the chronic phase, parasitological detection methods have very low sensitivity, whereas molecular detection can only be done in reference laboratories. Besides, clinical evaluation may not be specific to Chagas disease and cannot be used in cases where some structural tissue damage already exists. In addition, measuring seroconversion by conventional tests is not viable as it may take years or decades for a patient with chronic disease to revert serologically. Finally, the posttreatment detection of circulating parasites (through their DNA) by molecular amplification techniques, such as quantitative polymerase chain reaction (qPCR), may be useful for determining treatment failure, but a negative qPCR result cannot be considered a surrogate of cure [18].

Development of a test that can determine in a timely manner if a patient treated for Chagas disease has successfully responded to treatment has therefore been identified as a priority [16]. As mentioned above, such a test could be used in two different scenarios or use cases: (1) the daily clinical management (DCM) of Chagas disease patients posttreatment to decide if and/or when a patient should be followed up after treatment completion and (2) in the context of clinical trials (CT), where the test would be used as the endpoint measurement for the evaluation of new anti–*T*. *cruzi* treatments.

The development of this test (or tests) should be guided by a TPP. TPPs for a test to assess treatment response in Chagas disease patients have been suggested previously [17, 19]. Building on them, we now present a TPP specifically describing the required technical and performance characteristics of a test to determine if a Chagas disease patient has been cured posttreatment. We have considered two use scenarios: day-to-day healthcare provision and clinical evaluation of new anti–*T*. *cruzi* drugs or alternative regimens of the drugs currently available.

## Methods

As in previous TPPs [17, 19], we defined the test characteristics on the basis of Chagas disease expert opinion on the response to anti–*T*. *cruzi* treatment in Chagas disease patients. Discussions leading to this TPP document were coordinated and developed by the NHEPACHA (new tools for the diagnosis and evaluation of Chagas disease patients) network [20]. Created in 2011 with the goal of identifying and validating the use of biomarkers for Chagas disease, the network currently consists of 14 groups, 11 of them from America and the remaining three from Europe. The network includes expert clinicians working with patients, researchers working in academia, and specialists in industry and product development partnerships (PDPs). All of them were first asked to come to a consensus on a series of definitions to be used in the TPP (Table 1). Then they were asked to agree on the parameters for each of the test characteristics. The categories used in the TPP were adapted from previously published TPPs for diagnostic tests [21–23] and included specific features such as number of samples or timing of sampling.

Several face-to-face meetings were organized (in March 2017, March 2018, and March 2019), and email surveys were sent around in preparation for the final consensus document. For each of the characteristics in the TPP, specialists were asked to take into consideration both use-case scenarios. Since the requirements for a test to be used as an endpoint in clinical trials for new drugs or new regimens (use case 1; CT) may be more stringent, such a test should meet, in general, “ideal” conditions. This does not apply to the “operational characteristics” in which “ideal” conditions are related to a test to improve daily clinical management of treated Chagas disease patients (use case 2; DCM).

## Results

The TPP for a test for early assessment of treatment response in Chagas disease patients is presented in Table 2. This takes into consideration the following parameters: scope, performance, and operational characteristics.

**Table 2:** TPP for a test for early assessment of treatment response in Chagas disease patients.

Characteristic	Ideal	Acceptable	Comments
**Scope**			
Goal of test or intended use	To be used as an endpoint in CTs evaluating new anti–*T*. *cruzi* treatments or regimens.	To guide the management of Chagas disease patients posttreatment.	Objective: Develop a test to determine if a patient treated for Chagas disease has successfully responded to treatment, which is simple to perform and can be used as early as possible.
Target population to be tested	• Treated patients in the acute phase of infection (all types*). • Treated patients in the chronic phase of infection more than 1 year of age (all clinical forms**).	Treated patients in the chronic phase of infection >1 year of age, with an indeterminate clinical form or early tissue damage involvement (e.g., Kuschnir scale grades 0–1).	*Congenital, oral, reactivation upon immune-suppression, vector-transmitted. ** Indeterminate, cardiac, digestive, and cardio-digestive.
Level of implementation in the healthcare system	Healthcare structures with low-complexity laboratory facilities (i.e., equipped at most with an ELISA reader).	Healthcare structures (same level as where treatment is provided) with middle-to-high laboratory facilities (i.e., those with a quality-control program installed).	Here, the ideal conditions for the test would better suit the acceptable scenario (DCM rather than CT). Clinical trials are well-funded and rely on well-equipped facilities to run the required tests, whereas in most endemic settings it is common to have poorly equipped facilities.
Intended end-users	Healthcare workers with no laboratory skills.	Healthcare workers with laboratory training.	Here, the ideal conditions for the test would better suit the acceptable scenario (DCM rather than CT).
**Performance**			
Diagnostic sensitivity (Se)	Sensitivity equal or better than 95%, so that the test should be able to detect more than 95% of the patients in whom the treatment was efficacious.	Sensitivity equal or better than 60%, so that the test should be able to detect more than 60% of the patients in whom the treatment was efficacious.	Sensitivity for Chagas disease therapeutic efficacy (as defined above) means correctly identifying subjects in whom the treatment was efficacious. The sensitivity threshold established for each scenario should be included in the 95% CI.
Diagnostic specificity (Sp)	100%	More than 90%	Specificity for Chagas disease therapeutic efficacy (as defined above) means correctly identifying subjects who failed to respond to the treatment, so that they can be managed accordingly. The specificity threshold established for each scenario should be included in the 95% CI.
Geographic working range	Pan–*T*. *cruzi* test.	Test works in a particular region but not in all.	Eco-epidemiological geographic differences observed in Chagas disease are associated with the distribution of DTUs. In the ideal use-case scenario the test should be universal, i.e., capable of detecting all human-infecting lineages. In the acceptable use-case scenario, the test should work in at least one of the regions defined by Miles et al. [26].
**Operational characteristics**			In this section the ideal conditions for the test would suit the DCM scenario, whereas the acceptable condition would better suit the CT scenario.
Type of test	Single biomarker-based test.	Single or multiple biomarker-based test.	
Type of analysis	Qualitative.	Semiquantitative or quantitative.	
Format	Easy-to-use rapid test (e.g., lateral-flow immuno-chromatographic strip format).	Lab-based test (e.g. ELISA-type assay).	
Reading system	Visual—no instrument required.	Electronic-reader device required. Portable device preferred.	
Manual preparation of samples (steps needed after obtaining sample)	Maximum one step; precise volume control and timing may be required.	Several steps; precise volume control and timing required.	
Reagent integration and storage	All reagents should be contained in a single device. Reagent distribution and storage without cold chain.	External reagents may be needed and if required, should be included in the test kit, preferentially presented in a ready to mix, ready to use format. Reagents distribution and storage without cold chain.	All reagents and/or components of the kit must be available commercially.
Time to results (excluding sample collection)	Less than 3 hours.	Less than 24 hours.	
Type of specimen	Capillary whole blood (finger prick sample), saliva, and/or urine.	Whole blood extracted by venous puncture.	If blood samples are needed, finger prick samples would be preferred to venous extraction of blood. However, it must be considered that volumes larger than 50 μL will require venous puncture. It must also be considered that tests involving the use of sera will require a centrifugation step to segregate it from other blood components. This will require the availability of a centrifuge, which might not be the case in low-complexity laboratories.
Sample volume	Maximum volume by finger prick for rapid tests can be 50 μL.	Maximum volume: 5 ml in adults; 1 ml in children.	
Number of samples	A maximum of two samples: one pretreatment and one posttreatment.	A maximum of three samples: one pretreatment and up to two posttreatment.	
Timing of sampling (of the first posttreatment sample)	Sampling within 6 months of treatment.	Sampling within 24 months of treatment.	
Power requirements	None (instrument free), minimal portable equipment, or minimum requirements (battery operated or electricity for a short time).	Standard operating currents with built-in UPS for utilization in locations with variable power.	The fewer the infrastructure requirements (i.e., power, water, skills), the more likely is that this test can be adopted at lower levels, such as in the community or in primary healthcare facilities.
Maintenance	No maintenance or minimum maintenance required by technically trained personnel or remote support.	Preventive maintenance once a year or after running more than 1,000 samples; only simple tools and minimal expertise required; include maintenance alert. Mean time to failure of at least 18 months.	A maintenance alert and records on duration of use are essential to ensuring proper functionality in settings where it is unlikely that the device will always be handled by the same person. It is essential that only simple tools and minimal expertise are necessary to carry out maintenance, given the number of devices likely to be in use.
Calibration	None required.	Remote or autocalibration.	
Operating temperature	Between 5 and 50°C at up to 90% relative humidity.	Between 5 and 40°C at up to 70% relative humidity.	High environmental temperatures and high relative humidity are often present in countries where Chagas disease is endemic.
Operating altitude	Any altitude (up to 5,000 m).	Up to 4,000 m.	Andean regions above 3,500 m are not highly endemic for Chagas disease, but taking La Paz as an example (3,640 m), the minimal working altitude for the test should be established at this height.
Additional supplies (not included in the kit)	None. If required, supplies should be included in the test kit in a ready to use format.	If required, supplies should be easy to obtain, and preferentially presented in a ready to use format.	In the case of molecular biomarkers, the inclusion of low-cost equipment for nucleic acid extraction from collected samples should be considered. Otherwise, the sensitivity of the test might be compromised.
Internal quality control	Internal full-process positive controls and negative controls.	Internal full-process positive controls. In the case of molecular methods, negative controls would be also mandatory.	In addition to EQA.
Training and education needs	Less than 5 days of training.	Less than 6 weeks of training, laboratory personnel (biochemists, microbiologists).	Low training and education needs are desirable, but this will depend on the type of test (e.g., rapid diagnostic tests may require less training than laboratory-based assays). An EQA to survey the process and training should be included at least once a year.

CT, clinical trial; DCM, daily clinical management; DTU, discrete typing unit; ELISA, enzyme-linked immunosorbent assay; EQA, external quality assessment; TPP, target product profile; UPS, uninterruptable power supply

## Discussion

A test allowing the early assessment of antiparasitic treatment efficacy in Chagas disease patients has been recognized as a priority for a long time [16, 17, 19, 27]. However, despite some recent advances [28, 29], these tests are currently only available for research use.

The development and evaluation of such a test is challenging for multiple reasons, including the lack of a practical gold standard and consensus on the definition of a cure for Chagas disease patients (see Table 3). In this paper, we have tried to address these issues and built on the previous TPPs [17, 19] to develop a detailed description of the requirements of a ToC for two use cases: management of Chagas disease patients and development of new drugs or alternative regimens using currently available drugs.

**Table 3:** Challenges towards the development and evaluation of a test for the early assessment of treatment efficacy in Chagas disease patients.

Challenge	Description
Definition of cure	We agreed on parasite elimination as a surrogate of cure (see also the definition in Table 1). We acknowledged the difficulty to ensure that the parasite has been completely eliminated from the patient´s body. Nonetheless, assuming that it is the presence of the parasite that drives (1) the appearance of pathogenic events, (2) relapsing episodes, (3) and a long-term steady state of antiparasitic antibodies, then any future ToC must support such elimination. At the same time, we could have been taken into consideration clinical improvement, but this would have ignored a large proportion of chronically infected asymptomatic people undergoing treatment. Moreover, evaluation of clinical improvement requires another type of test that can unequivocally show tissue damage due to Chagas disease and its improvement upon treatment.
Lack of (applicable) gold standard	The current standard to determine cure is serological reversion from positive to negative in two conventional tests based on distinct antigen sets [4, 5]. With this reference, variable cure rates of 8%–40% have been reported in adult patients treated in the chronic stage who were followed for 10 to 20 years [13, 30]. However, this is impractical from any perspective, whether the daily management of the disease or the performance of a clinical trial, because average follow-up periods do not last that long.
Lack of well-characterized samples from patients	There is a limited number of samples from Chagas disease patients that include baseline and follow-up samples collected over decades; such samples would accelerate the identification of new biomarkers and the evaluation of tests to assess treatment efficacy. Availability to the scientific community of these samples will be fundamental for the development of much awaited tests for the early assessment of treatment response.
Quantitative test	What constitutes a significant change in biomarker levels should be determined, whether it be by serological evaluation of the immune response to a parasite or host-derived antigen or the measurement of a molecular-based readout.

ToC, test of cure

This TPP should guide the development of tests to rapidly evaluate Chagas disease antiparasitic treatment efficacy. These tests might be based on biomarkers derived from the parasite, such as PFR2, KMP11, HSP70, the peptide 3973, F29, αGal-containing antigens, and the list of epitope-based antigens provided by Granjon and colleagues [28–34]; biomarkers derived from the host, such as hypercoagulability markers F1+2 and ETP [35], and the APOA1 and FN fragments [36]; or a combination of both. At present, preliminary results using Infinity antigen 3 (AG 3; derived from the parasite) and the SaMi-Trop cohort from Brazil show promise, but further insight is required to ensure that the 40% parasite clearance reported upon treatment persists over time [28]. It also remains to be shown how this compares to trends in conventional serology reactivity and whether similar levels of response can be found with samples from other geographic origins [28]. All the studies that evaluated host-derived markers were performed with a reduced number of samples, and therefore their potential will need to be assessed with larger collections.

The TPP can also help to evaluate the approaches currently used to assess treatment efficacy: serology and qPCR. The latter has been used in clinical studies as a test for treatment failure. Serial blood sampling and molecular amplification reactions have been implemented to assess the absence of circulating *T*. *cruzi* DNA in chronically infected patients during treatment follow-up [37]. A major limitation of the use of qPCR to monitor treatment response is that it has not been assessed in long-term cohorts and studies; consequently, a negative molecular outcome at a specific time cannot exclude that a relapse may occur later on. As a result, there remains an urgent need for more reliable and straightforward tests to evaluate treatment efficacy, which we expect this TPP can help to streamline.

## Conclusion

We have presented a new and complete TPP for the development of tests for the early assessment of Chagas disease treatment efficacy. In the context of this neglected infectious disease, this is mostly an underrepresented area of investigation, and the current lack of such tests greatly hampers the management of patients and control of the disease.

Today, the large majority of the 6 to 7 million people infected by *T*. *cruzi* remain untreated [3]. Recent advances in diagnostics (e.g., use of rapid diagnostic tests) [38] and treatment (e.g., a shorter course—two weeks instead of eight weeks—of BNZ), as well as the implementation of new access strategies and an increasing availability of drugs, will hopefully result in a rapid increase in the number of patients treated in the next few years [15]. A test for the early assessment of treatment efficacy will be fundamental to managing those patients, as well as to accelerating the evaluation of new drugs or regimens. The TPP described in this article can guide the development and uptake of these tests.
